# Insights into the protective effect of omega-3 nanoemulsion against colistin-induced nephrotoxicity in experimental rats: regulation of autophagy and necroptosis via AMPK/mTOR and RIPK1/RIPK3/MLKL signaling pathways

**DOI:** 10.1080/0886022X.2024.2429686

**Published:** 2024-11-25

**Authors:** Medhat Taha, Omer Abdelbagi, Tourki A. S. Baokbah, Rehab M. Bagadood, Naif A. Jalal, Rami Obaid, Nawal E. Al-Hazmi, Naeem F. Qusty

**Affiliations:** a Department of Anatomy, Al-Qunfudah Medical College, Umm Al-Qura University, Al-Qunfudhah, Saudi Arabia; b Department of Anatomy and Embryology, Faculty of Medicine, Mansoura University, Mansoura, Egypt; c Department of Pathology, Qunfudah Faculty of Medicine, Umm-Al-Qura University, Al-Qunfudhah, Saudi Arabia; d Department of Medical Emergency Services, College of Health Sciences-AlQunfudah, Umm Al-Qura University, Al-Qunfudhah, Saudi Arabia; e Department of Clinical Laboratory Sciences, Faculty of Applied Medical Sciences, Umm Al–Qura University, Makkah, Saudi Arabia; f Department of Microbiology and Parasitology, Faculty of Medicine, Umm Al‐Qura University, Makkah, Saudi Arabia; g Department of Medical Genetics, Faculty of Medicine at Al-Qunfudah, Umm Al-Qura University, Al-Qunfudhah, Saudi Arabia; h Department of Chemistry, Division of Biology (Microbiology), University College of Qunfudah, Umm Al-Qura University, Al-Qunfudhah, Saudi Arabia

**Keywords:** Omega-3 nanoemulsion, colistin, nephrotoxicity, oxidative stress, autophagy, necroptosis

## Abstract

Colistin is considered one of the most effective antibiotics against gram-negative bacteria. However, nephrotoxicity is one of the dose-limiting factors in its treatment. This study aimed to evaluate the outcome of omega-3 nanoemulsion against colistin-induced nephrotoxicity and its possible underlying mechanism. Four rat groups were involved in the present research; each group containing ten rats was divided as follows: Group I (control) rats received normal saline; Group II (omega-3 nanoemulsion) rats received a dose of 500 mg/kg/body weight orally; Group III (colistin) rats received colistin intraperitoneally (300.000 IU/kg/day); and Group IV (colistin/omega-3 nanoemulsion) rats were treated for six days. The results revealed that colistin administration caused deterioration in renal functions such as creatinine, blood urea nitrogen, 24 h proteinuria, and kidney injury molecule-1 with decrease in creatinine clearance, resulting in histological alternation and tubular damage with diffuse interstitial inflammation. Additionally, colistin significantly increased the lipid peroxidation marker malonaldehyde, proinflammatory cytokines tumor necrosis alpha, interleukin-6, interleukin-1 beta. Also, autophagy influx marker microtubule-associated protein light chain 3B, Beclin-1, and necroptotic related proteins, receptor-interacting protein kinase-3 (RIPK-3), RIPK-1, mixed lineage kinase domain-like protein, and autophagy pathway regulatory kinase AMP-activated protein kinase, with a decrease in antioxidant enzymes catalase, superoxide dismutase, and total antioxidant capacity, autophagic marker ubiquitin-binding protein (p62), and regulator Mammalian target of rapamycin. Interestingly, omega-3 nanoemulsion reversed the results above, dramatically improving renal function and histological picture. Thus, omega-3 nanoemulsion provided a notable method for suppressing colistin-induced nephrotoxicity *via* its antioxidant and anti-inflammatory power, inhibiting pathological autophagy and necroptosis.

## Introduction

Colistin is a cyclic antibiotic with limited use due to its nephrotoxic effect [1]. Recently, multiple antibiotic resistances have been developed in intensive care patients. For this reason, colistin usage has increased due to its role in patients’ survival and clinical improvement. Colistin affects renal proximal tubules, disturbing their permeability and resulting in the passage of electrolytes and water. Its cytotoxic effect deteriorates renal function through elevations in creatinine, blood urea nitrogen (BUN) levels, hematuria, proteinuria, glucosuria, and acute tubular necrosis [2]. Colistin-induced nephrotoxicity is usually reversible [3]. Generally, laboratory renal values return to normal within one month [4]. Restricted application of nephrotoxic dose-dependent colistin forced the researchers to use nephroprotective drugs in combination with colistin.

Several studies have reported that oxidative stress and the accumulation of free radicals are associated with renal tubular apoptosis-mediated colistin-induced renal pathology [5]. Moreover, colistin induced severe renal inflammation, increasing renal damage [6]. Autophagy is a physiological process essential for cell survival characterized by lysosomal degeneration intracellular organelles [7]. It is well known that oxidative stress is considered one of the factors for the induction of the autophagy process [8]. Several studies on experimental renal damage have documented the beneficial role of autophagy, especially when it is kept within a specific range [9,10]. Reactive oxygen species induce autophagy by MAPKs, FOXO3, p53, Atg5, Atg4, NRF2, ER stress, and inhibition of the Akt/Mammalian target of rapamycin (mTOR) pathways, which initiate the autophagy process through the phosphorylation of Bcl-2, dissociating it from Beclin-1. The expression of Beclin-1 is indicative of the autophagic state of the cell. Furthermore, the microtubule-associated protein light chain 3B (LC3B) is central to the autophagic activity required to elongate phagophores [11,12].

Necroptosis is defined as programmed cell death resulting from stimulation of receptor interactive serine-threonine protein kinase one (RIPK-1) [13,14], with subsequent activation of receptor interactive serine-threonine protein kinase three (RIPK-3), which plays a role in phosphorylating a protein called mix lineage kinase domain-like (MLKL) with its translocation to the inner surface of the cell membrane. As a result of this sequence activation, it induces cell swelling and cell membrane damage, releasing damage-associated molecular patterns (DAMP) and an inflammatory reaction by cells. The RIPK3-MLKL complex was recently named the necrosome. Necroptosis, recently reported in acute kidney injury, resulted from ischemia-reperfusion (IR), sepsis, cisplatin, and cyclosporine-induced nephrotoxicity [15–18].

Omega-3 fatty acids are the famous polyunsaturated lipids of docosahexaenoic, eicosatetraenoic, and alpha-linolenic acids [19]. It is known that the human body not able to synthesize omega-3 fatty acids, so they should be supplied to the body externally [20]. The delivery of omega-3 in fish diets faces a severe problem due to their unsaturated nature, leading to the deterioration of their lipid properties [21]. Furthermore, omega-3 prevents several diseases owing to its anti-inflammatory and antioxidant properties [22,23]. Previous studies documented its ameliorative effect against lipopolysaccharide-induced renal injury [24]. Nanoemulsions (NEs) are stable thermodynamic dispersions of water and oil stabilized by a film of interfacial active agents [25–27]. NEs are usually prepared in the form of droplets with a size range from 100 to 1000 nm, which were fabricated in the form of water in oil, oil in water, and multiple emulsions in water/oil/water [28]. Nanoemulsion preparation presented several advantages when compared to conventional emulsions, including its ability to incorporate different hydrophobic and hydrophilic drugs, large surface area helps to localize therapeutic drugs on the target site with minimization of side effects, improved bioavailability and drug solubility, sustained drug release [29,30]. The current study aimed to formulate omega-3 in nanoemulsions, facilitating their delivery. In addition, it aimed to examine its novel impact against colistin-induced nephrotoxicity, exploring its mechanistic role in reducing autophagy-mediated necroptosis and improving tubular epithelial health.

## Material and methods

### Preparation of omega-3 nanoemulsion

Omega-3 was bought from Sigma Company, USA. A solitary layer of omega-3 nanoemulsion oil in water was produced [30]. Omega-3 nanoemulsions were prepared by slowly dropping around 10 mL of surfactant tween 80 in a magnetic stirrer at room temperature. The dropping flow is fixed at 1 mL per minute. Then, the nanoemulsion was dispersed over an ultrasonic bath for half an hour and homogenized by using an ultrasonic probe integrated with a homogenizer (Sonics Vibra-cell^™^, Model VC 505, Inc., Newtown, CT, USA) under the ulterior conditions: timer, 5 min; amplitude, 60%; and pulser, 1 s ON/1 s OFF to obtain omega-3 nanoemulsion.

### Omga-3 nanoemulsion characterization

An electron microscope (TEM, JEOL JEM-2100, Tokyo, Japan) was used to screen the inner morphology of the newly made omega-3 nanoemulsion at 200 kV. The picture capture analysis procedure used the Digital Micrograph and Soft Imaging Viewer software (Gatan Microscopy Suite Software, version 2.11.1404.0). The mean vesicular sizes (Z-average) of the nanoemulsion, the external charge of the nanoemulsion particles (Z-potential), and the polydispersity index (PDI) were measured using a Zetasizer Nano ZS analyzer.

### Experimental animals

Forty Sprague-Dawley male rats weighing about 200–230 g were kept in chain network confines in a room that was 80% humid and with temperatures ranging from 22 to 26 °C with equal light and dark cycles. Standard water and food were given regularly. This study was approved by the Scientific Research Ethics Committee of Umm Al-Qura University, Makka, Saudi Arabia with approval number (HAPO-02-K-012-2023-11-1878), in accordance with Animal Research Reporting of *in Vivo* Experiments (ARRIVE) guidelines. Moreover, all experimental procedures and research protocols were performed in accordance with the guidelines and regulations laid down and duly approved by Scientific Research Ethics Committee of Umm Al-Qura University.

### Study design and tissue sampling

The 40 rats were divided into four groups: Group I (the control group): rats received saline by oral gavage for six consecutive days. Group II (omega-3 nanoemulsion group): rats received omega-3 nanoemulsion with a 500 mg/kg b.wt. dose through oral gavage [31]; Group III (Colistin Group): rats received colistin from sigma Aldrich (ST, Louis, MO, USA) for six days at 300.000 international units per kilogram daily. This dose proved nephrotoxic, according to a previous study by Ozyilmaz et al. [32]. Group IV (colistin + omega-3 nanoemulsion): rats received intraperitoneal colistin (300,000 IU/kg/day) and omega-3 nanoemulsion for six days. At the end of the study, rats were housed in different cages for a whole day to collect urine samples for measuring total protein and kidney injury molecule-1 (KIM-1). After that, the rats were exposed to intraperitoneal anesthesia using 150 mg/kg of sodium pentobarbitone, followed by cervical dislocation. Blood was withdrawn from the retroorbital venous plexus. The serum was separated and stored at −20 °C for subsequent measurement of the renal profile. Two slices were taken from the left kidney of each group. The first slice was homogenized in cold phosphate-buffered saline and centrifuged at 3000x. A supernatant was used to measure oxidative stress markers. The second slice was stored at −80 °C for molecular analysis. Right kidneys were fixed in 10% formalin for routine histologic and immunohistochemistry examinations (LC3B, p62, p-MLKL, RIPK-1).

### Biochemical parameters

#### Renal function assessment

After mercy killing, the serum was immediately put into 1.5 mL polypropylene tubes and stored at −20 Celsius for assessment of kidney function tests *via* colorimetric testing, using the blood urea nitrogen and creatinine identification kits (cat number URE 118100 and CRE 106100 for urea and creatinine respectively) bought from bio diagnostic company Egypt and performed per the manufacture direction. Twenty-four hours urine sample were analyzed, for KIM-1 protein to assess renal damage, using a commercial ELISA kit (cat RKM29-KO1). Additionally, 24-h urinary protein level were done. Moreover, creatinine clearance (Cr Cl) was estimated as follows:

$$\text{Cr Cl }(\text{ml}/\text{min})=\frac{\text{Urine volume }(\frac{\text{ml}}{24hr})x\;\text{Urine creatinine concnetration}(\frac{\text{mg}}{\text{dl}})}{\text{plasma creatinine}(\frac{\text{mg}}{\text{dl}})}$$

#### Renal oxidative stress marker assessment

Investigating oxidative stress markers malonaldehyde (MDA), total antioxidant capacity (TAC), catalase (CAT), and superoxide dismutase (SOD) of renal tissue supernatant by commercial kits (Biodiagnostics Co., Cairo, Egypt) according to the manufacturer’s protocol in the production factory.

#### Microscopic examination

The right kidney tissue was filled with 10% formalin for two days. Then, the tissue was put in a processing machine. Dehydrated by immersing in ascending concentrations of ethanol (70%, 80%, 90%, and 100%), cleared with xylene, followed by wax impregnation. After that, it was embedded in the paraffin block. Five µm of paraffin sections of renal tissues from different experimental groups were deparaffinized by xylene for 45 min, rehydrated with an descending scale of alcohol (100%, 96%, and 80%), and then stained with hematoxylin and eosin. Blinded pathologists examined the different sections in experimental groups. histopathological scoring for kidney according to Zheng et al. [33] with little modification (Table 1).

**Table 1.** Lesion score: criteria for histopathologic scoring of rat kidney.

Score	Tubular damage	Inflammation	Fibrosis
0	None	None	None
1	Minimal, few	Few, rare	Few
2	Mild to moderate tubular degeneration with few intraluminal cast	Mild, focal	Moderate interstitial inflammation
3	Diffuse, many tubular damages with intraluminal cast	Moderate to severe coalescing interstitial inflammatory aggregates	Severe interstitial fibrosis

#### Immunohistochemical investigation

Renal tissue sections with 5 µm thickness were deparaffinized with xylol and rehydrated with ascending scales of ethanol. Then, to block the endogenous peroxidase, it was incubated for 10 min in 0.1% hydrogen peroxide, then incubated with the primary antibodies anti-RIPK1 (Cat# ab72139, Abcam, 1:00), anti-LC3B (Cat# PA1-16930, 1:200), anti-Phospho-MLKL (Cat# PA5-105678, 1:100), and anti-p62 (ab91526, Abcam, 1:100). To continue the immunostaining process, the slides were incubated with secondary anti-rabbit antibody kits for 30 min with a dilution of 1:200. Lastly, slides were incubated with a chromogen called diaminobenzidine (DAB). A counterstain of hematoxylin was used [34]. ImageJ software was used for each renal tissue region to perform a positive area staining mean (calculated by averaging data from 10 fields at 10-x magnification).

#### ELISA assay for TNF-α. IL-6, IL1β, P-AMPK, mTOR, beclin-1, and ULK1

Tumor necrosis factor-alpha (TNF-α). Interleukin-6 (IL-6), IL1β, P-AMPK, mTOR, beclin-1, and ULK1 levels were measured in tissue from kidney homogenates using ELISA kits (Cat# 88-7340-88, SEA079Ra, ERIL1B, ER0730, MBS7254602, MBS2706719, and MBS9354404) as per the instructions provided by the manufacturer. Standard and samples were placed on a 96-well plate, coated with an adhesive strip, and mainlined for two hours at 37 degrees Celsius. Liquids were extracted from all wells without washing. Biotin-linked antibody was added to each well coated with a fresh adhesive strip and incubated at 37 Celsius for 60 min. Then, the antibody was eliminated, and the well was washed 3 times with a supplied puffer Avidin-HRP. The microtiter plate was then covered with a fresh strip and incubated for 60 min at 37 degrees Celsius. The mixture was then removed, and the well was rinsed finely. The substrate made from TMB was put in each well and incubated for twenty minutes at 37 degrees Celsius in a dark place. STOP solution was added to each well, and the optical density was detected at 450 nm using a microplate reader.

#### Real-time PCR analysis

The total RNA was measured from renal tissue homogenate by direct-Zol RNA (Cat #R 2072, United States). RT -PCR (Cat #EP0451) (Thermo Fisher Scientific, MA USA) was used to transcript total RNA into complementary DNA (cDNA), StepOnePlus real-time PCR (Applied Biosystems, USA) was used for the thermal profile. The data were obtained at the cycle threshold (Ct) for housekeeping and target genes. Receptor-interacting protein kinase 3 (RIPK-3), AMP-activated protein kinase (AMPK), and mammalian target of rapamycin (mTOR) were performed as per critical threshold mean (CT) expression. The GAPDH gene and the relative quantitation of target genes were measured by the 2^ΔΔCt^ method. The primers for RIPK3 forward as 5′-TTAAACGCGAAGGCAACGA-3′ and reversed as 5-’ CAGTCTCCTCCTGCTGTCGAT-3′ (the bank number of the gene XM_032898971.1); AMPK forward as 5′-AGCTCGCAGTGGCTTATCAT −3′ and reversed as 5′- GGGGCTGTCTGCTATGAGAG −3′. (the bank number of the gene NM_023991.1); mTOR forward as 5′-ACGAAGGAGACAGACCGAAG −3′ reversed as 5′-CGACGAAGTCACTAGATTCA −3′ (the bank number of the gene AM_943028.1); GAPDH housekeeping forward as 5′- CCTCGTCTCATAGA-CAAGATGGT −3′ reversed as 5′- GGGTAGAGTCATACTGGAACATG −3′ (the bank number of the gene NM_001394060.2).

#### Statistical analysis

All data were statistically analyzed using GraphPad Prism software (version 8.0; La Jolla, CA, United States). They will be estimated means and standard deviations for each variable. Differences between means of different groups are performed using one-way ANOVA followed by posthoc Tukey tests, with a significant difference if the p value is statistically lower than 0.05.

## Results

### Omega-3 nano emulsion character

**Figure 1.**
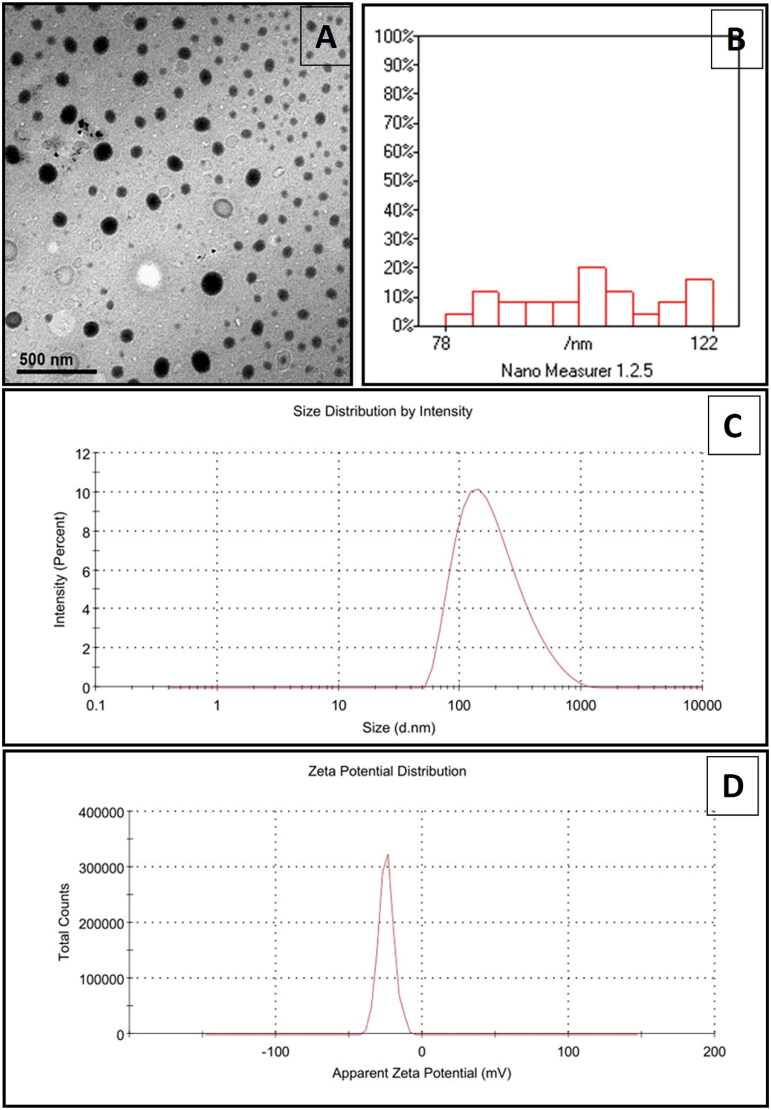
(A) The characterization of omega 3-nanoemulsion by TEM (type JEOL-JEM 2100). (B) Frequency distribution, which shows the distribution of nanoparticle sizes across different size ranges, along with descriptive statistics like the mean size and frequency percentages. (C) The Zeta size distribution by intensity. (D) The Zeta potential distribution.

The characterization of omega 3-nanoemulsion by TEM (type JEOL-JEM 2100) revealed its spherical morphology with minimal or no aggregation, with regular distribution of the size of nanoparticles size (Figure 1A,B). The average Zeta size distribution of omega 3 nanoemulsion presented a mean value of 150.4 nm in diameter (Figure 1C). PDI is 0.21 nm. Zeta potential dispersion has a mean value of −24 mV (Figure 1D).

### Effect of omega-3-loaded nanoemulsion on colistin-induced renal function deterioration

**Figure 2.**
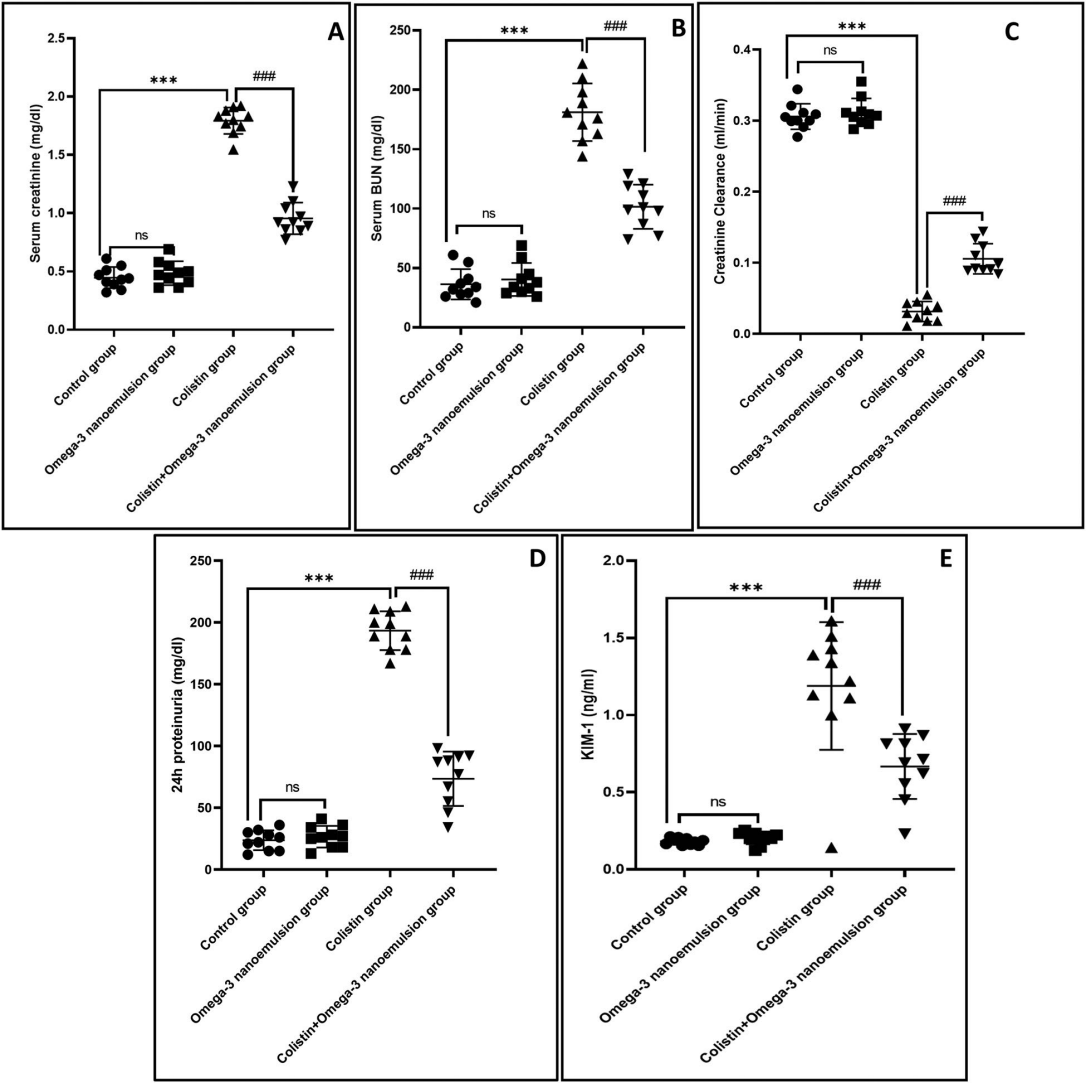
The effect of omega-3 nanoemulsion on renal function tests for creatinine, BUN, creatinine clearance (CrCl), 24 h proteinuria, and KIM-1. Values are displayed as standard deviation and mean ***p < 0.001 versus control group ^###^*p* < 0.001 versus colistin group.

Colistin administration significantly *p* ˂ 0.001 elevated renal function tests creatinine and BUN, 24h proteinuria as well as KIM-1 by 1.79 ± 0.11, 181.1 ± 24.2, 193.3 ± 15.71and 1.18 ± 0.41, respectively, and decreases creatinine clearance by 0.03 ± 0.01 compared to control groups (Figure 2A–E). Fortunately, oral intake of nanoemulsion significantly *p* ˂ 0.001 decreased renal function by 0.95 ± 0.13, 101.6 ± 18.61, 73.5 ± 22.00 and 0.66 ± 0.21, with increase in creatinine clearance by 0.10 ± 0.02 in relation to colistin group. The finding mentioned above reported its nephroprotective effect against colistin-induced nephrotoxicity.

### Antioxidant effect of nanoemulsion against colistin-induced oxidative stress

**Figure 3.**
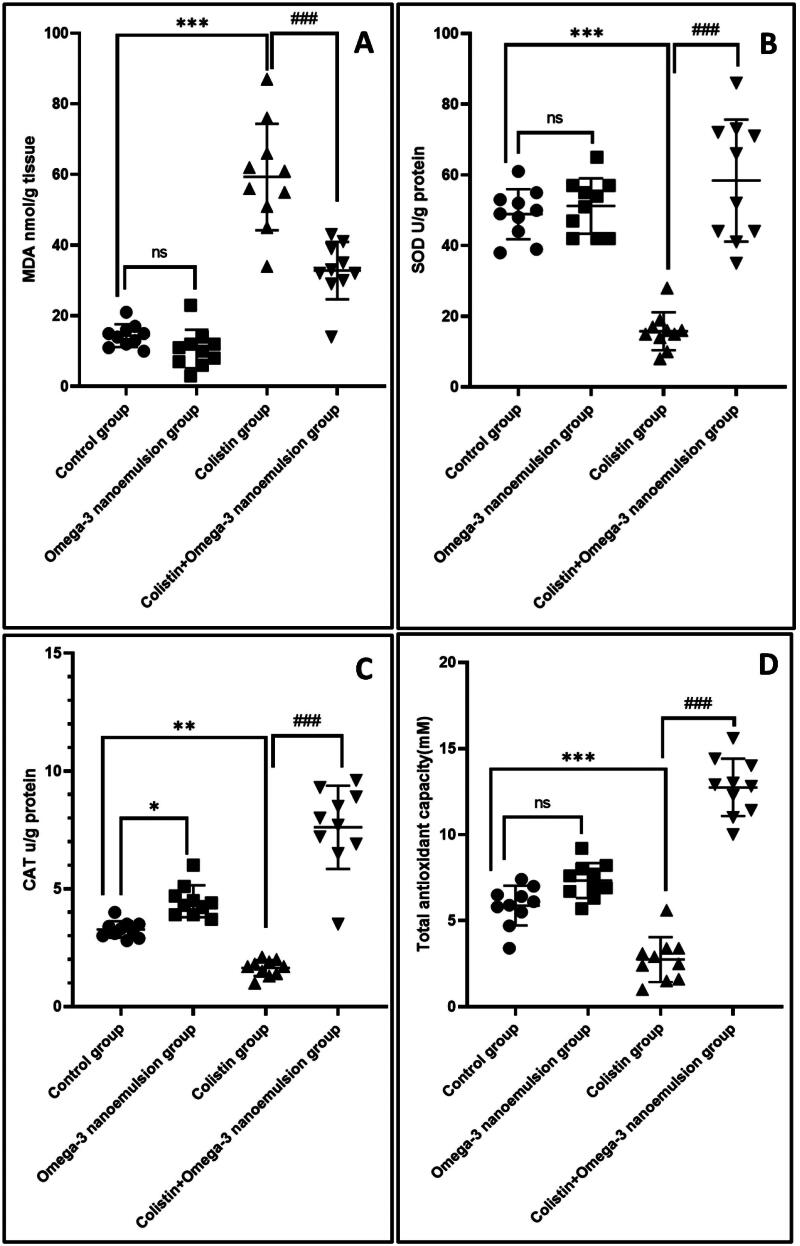
Homogenate renal oxidative markers MDA, SOD, CAT, and TAC. Values are displayed as standard deviation and mean ***p < 0.001 versus control group ^###^*p* < 0.001 versus colistin group.

Intraperitoneal injection of colistin significantly *p* ˂ 0.001 induces renal oxidative insult by increasing lipid oxidation markers MDA and decreasing antioxidant markers SOD, CAT, and TAC by 59.30 ± 15.7, 15.80 ± 5.37, 1.64 ± 0.34, and 2.73 ± 1.30, respectively. In contrast, omega-3 nanoemulsion intake with colistin markedly decreased renal homogenate MDA by 32.80 ± 8.10. It increased SOD, CAT, and TAC by 58.40 ± 17.26, 7.61 ± 1.77 and 12.74 ± 1.67 (Figure 3A–D). In Figure 3C,D, omega-3 nanoemulsion treatment tends to upregulate CAT and TAC in comparison to the control group. The antioxidant character of nanoemulsions is apparent.

### Effect of omega-3 nanoemulsion on renal histology

**Figure 4.**
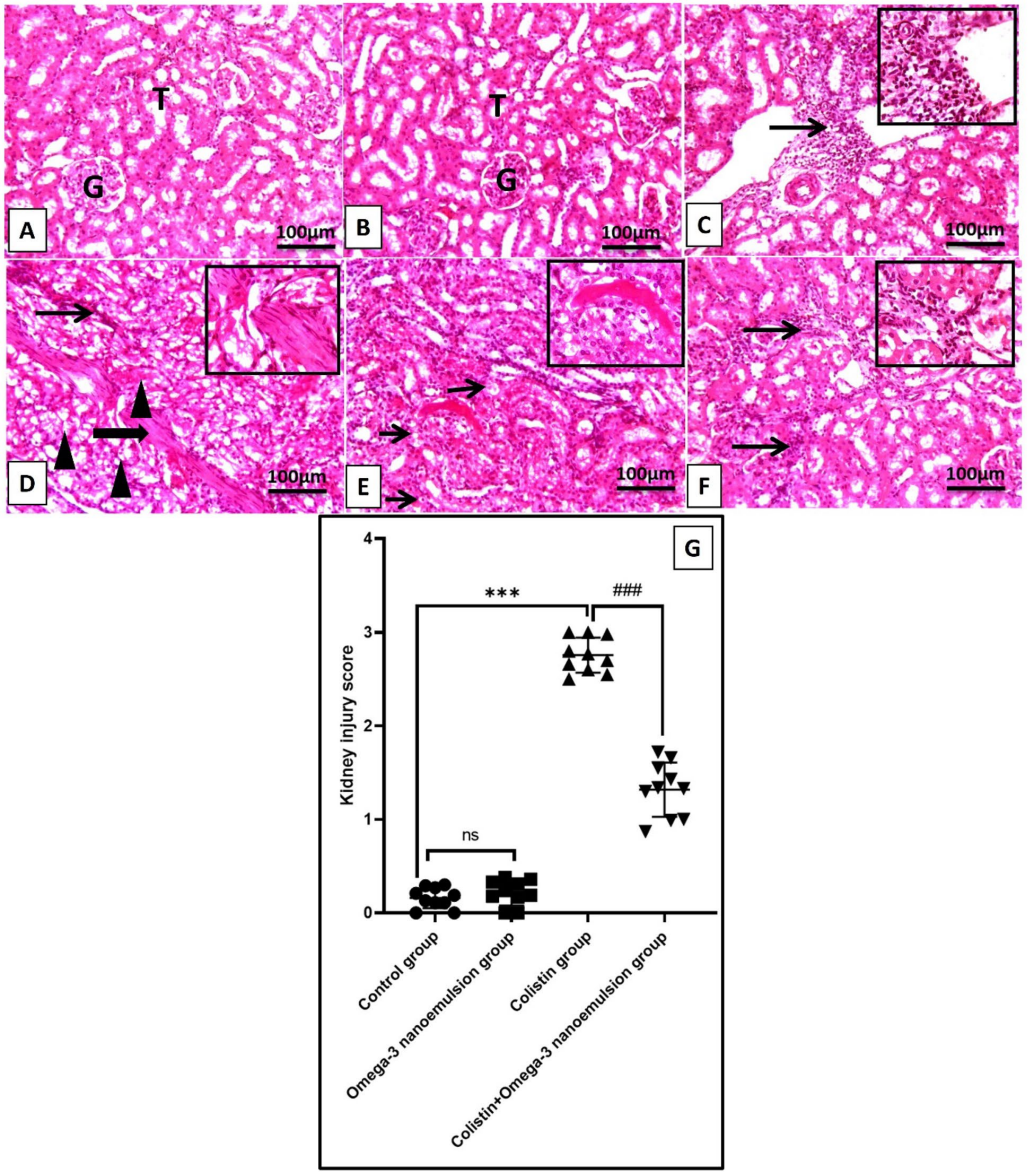
Representative photomicrographs of kidneys from different treatment groups. (A, B) The control and omega-3 nanoemulsion groups (*n* = 10) show a normal histological appearance of renal architecture (tubular (T) and glomerular (G) architecture). (C) The colistin group (*n* = 10) shows a severe interstitial inflammation; replaced and separated renal tubules with many mixed cellular infiltrates (thin arrow); inset, the inflammatory infiltrates comprised lymphocytes, macrophages, and fibroblasts. (D) The colistin group (*n* = 10) reveals focal, coalescing interstitial fibrosis (thick arrow) admixed with inflammatory cells (thin arrow); the adjacent tubules show diffuse degenerative and necrotic changes (arrowheads), and inset, a thick band of fibrous tissue admixed with a few lymphocytes replaced and surrounded by a degenerated tubule. (E) The colistin group (*n* = 10) demonstrates tubular hydropic degeneration (thin arrow) and inset hydropic degeneration in a degenerated tubule. (F) Colistin + omega-3 nanoemulsion (*n* = 10) shows focal to multifocal periglomerular inflammatory aggregations (thin arrows); inset aggregation of lymphocytes, macrophages, and fibroblasts surrounded by a mildly necrotic tubule. (Image magnification = 100x, inset = 400x. (G) Histogram of kidney injury score, Values are displayed as standard deviation and mean ***p < 0.001 versus control group ###*p* < 0.001 versus colistin group.

Histological examination of control and nanoemulsion renal tissues revealed a typical histological picture of renal glomeruli and tubules (Figure 4A,B). Meanwhile, H&E examination of renal tissues of colistin-treated rats showed a marked picture of nephrotoxicity in the form of diffuse interstitial inflammation with infiltration of macrophages, lymphocytes, and fibroblasts, regarding the tubules; it demonstrated severe tubular hydropic degeneration (Figure 4C–E). On the controversy, rats in group IV that received colistin and nanoemulsion showed clear histological improvement in the form of focal interstitial inflammation with a few necrotic tubules (Figure 4F). This result is supported by a significant P ˂ 0.001 decrease in kidney injury score compared to the colistin group (Figure 4G). The improvement of nanoemulsions in renal morphology explained their positive effect on renal function tests.

### Anti-inflammatory effect of omega-3 nanoemulsion against colistin-induced nephroinflammation

**Figure 5.**
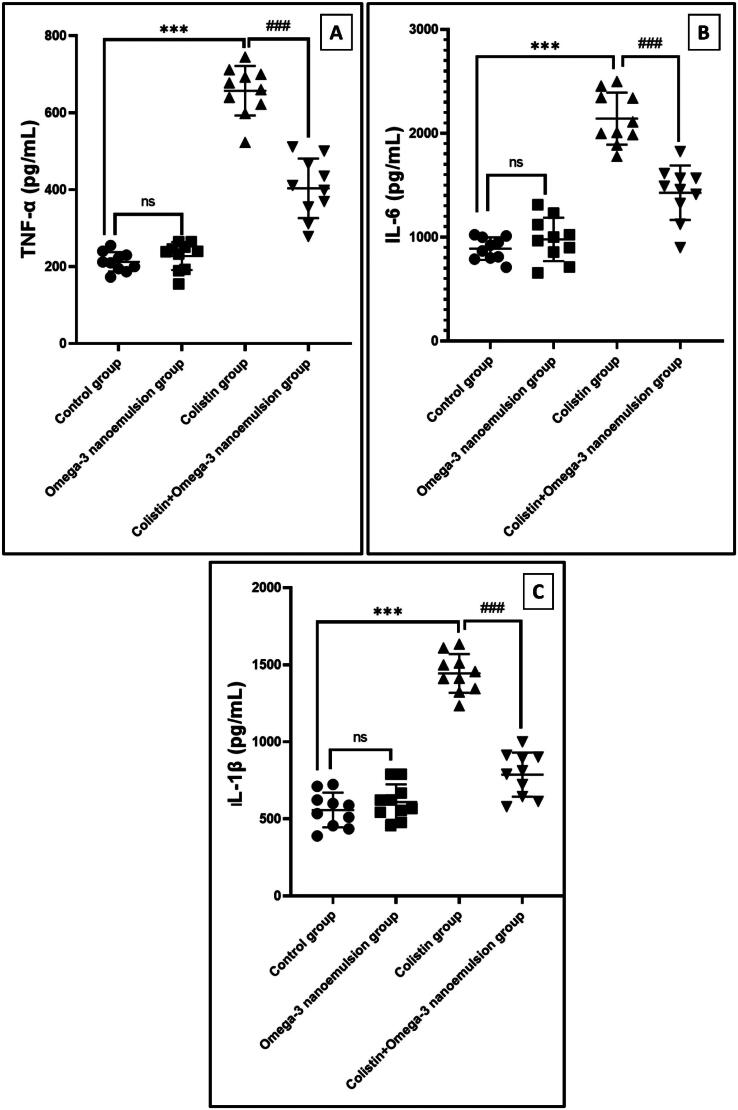
The effect of the omega-3 nanoemulsion on TNF-ά, IL-6, and IL-1β (A, B, and C). Values are displayed as standard deviation and mean ***p < 0.001 versus control group ^###^*p* < 0.001 versus colistin group.

Elisa assays of TNF-α, IL-6, and interleukin-1 beta (IL-1β) in the renal supernatant of the colistin rats group revealed a significant *p* ˂ 0.001 elevation in its level by 657.1 ± 64.39, 2142 ± 250.6, and 1444 ± 125.6, respectively, in comparison to control rats (Figure 5A–C). In addition, oral intake of omega-3 nanoemulsion with an intraperitoneal injection of colistin significantly *p* ˂ 0.001 decreased inflammation cytokines by 403.5 ± 77.34, 1427 ± 261.8, and 786.1 ± 143.1 concerning the colistin group. Based on this result, the omega-3 nanoemulsion exhibited a strong anti-inflammatory character against colistin-induced renal inflammation.

### Suppressive influence of omega-3 nanoemulsion on renal autophagy

**Figure 6.**
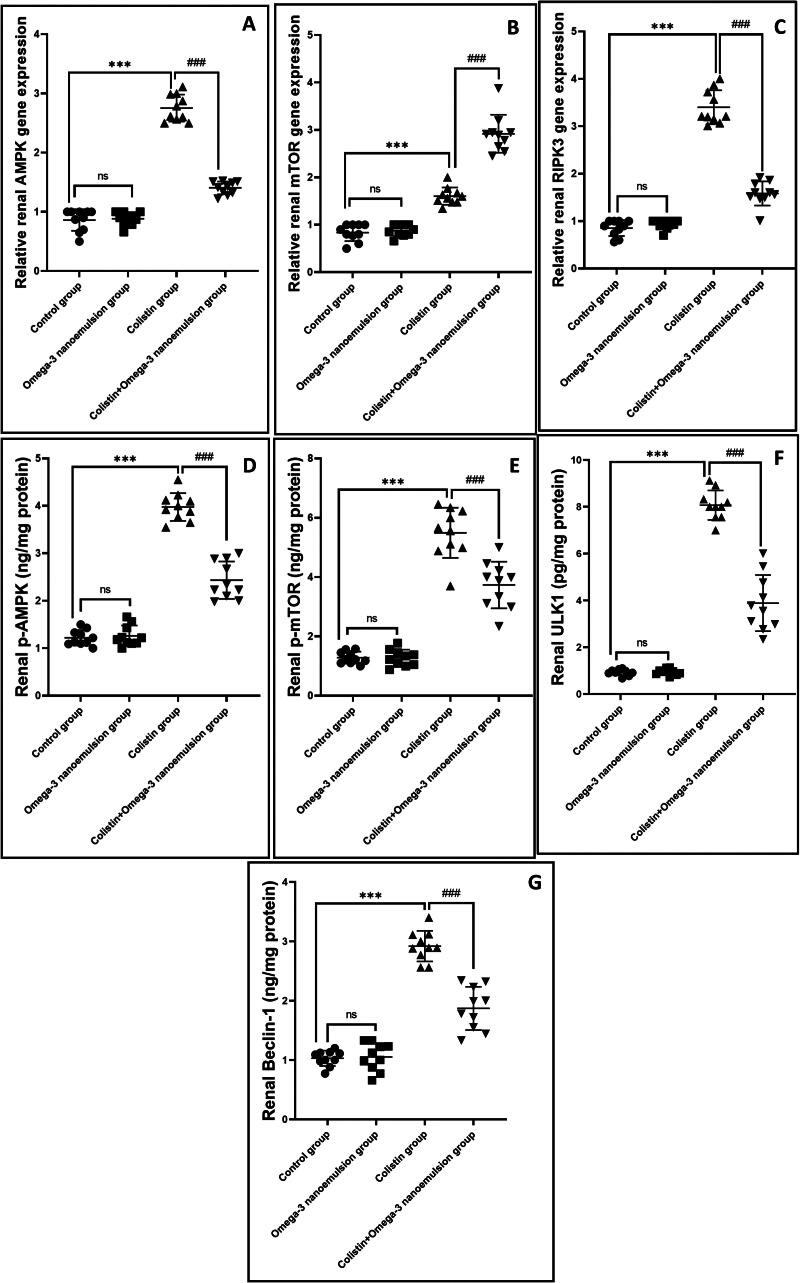
reveals the effect of omega-3 nanoemulsion on gene expression of autophagic gene regulators (AMPK, mTOR) (A, B) and necroptotic markers (RIPK3) (C), and protein level of p-AMPK, p- mTOR, ULK1 and Beclin-1 (D, E, F, G) in renal tissues of different experimental groups (*n* = 10). Values are displayed as standard deviation and mean ***p < 0.001 versus control group ###*p* < 0.001 versus colistin group.

**Figure 7.**
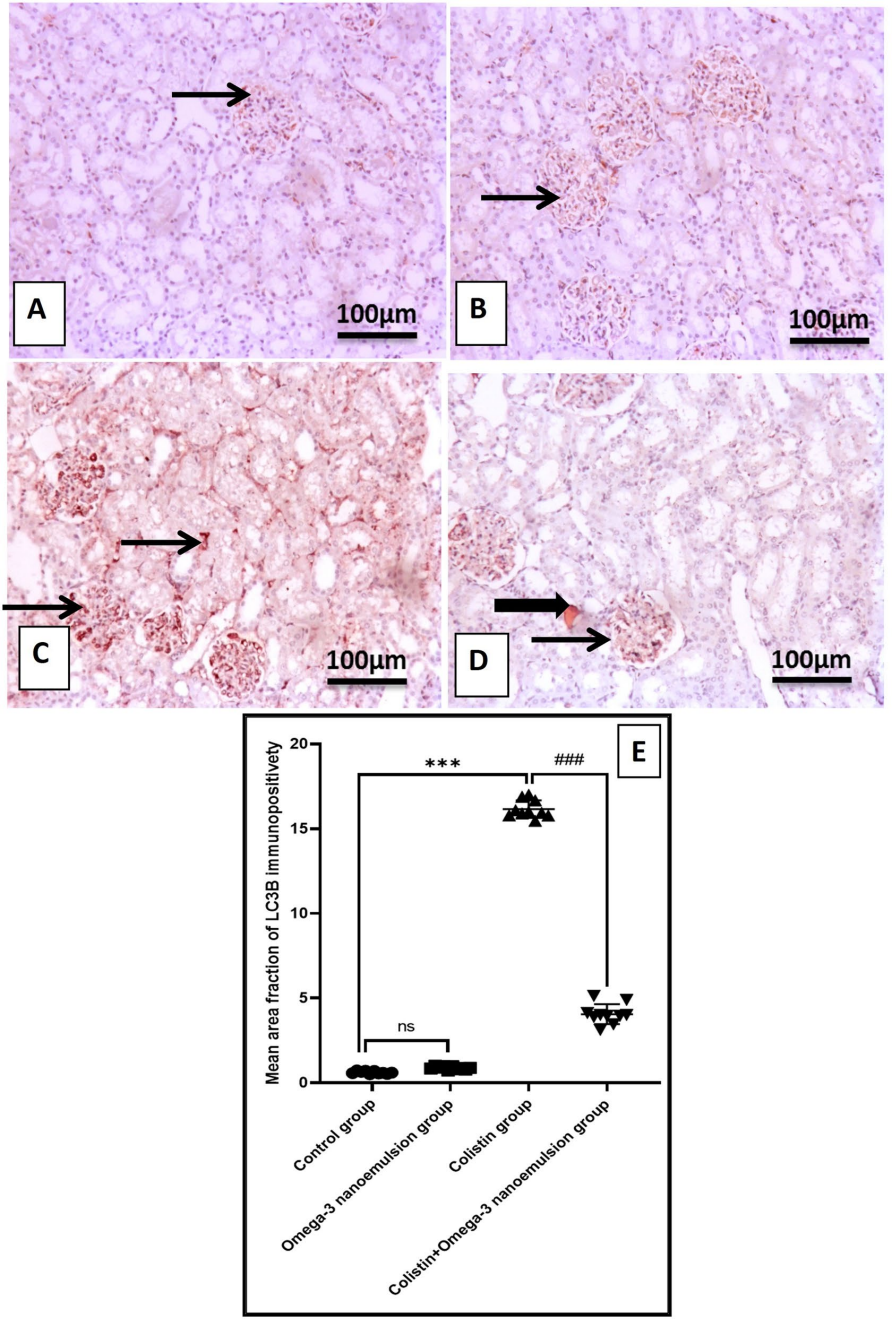
The representative IHC of LC3B expression in the kidneys of different treatment groups. (A and B) The control and omega-3 nanoemulsion groups (*n* = 10) display few expressions in glomerular tufts. (C) The colistin group (*n* = 10) show intense, strongly immunopositively stained glomerular tufts and tubular epithelial cells. (D) The colistin + omega-3 nanoemulsion group (*n* = 10) depicts mild glomerular and tubular immunopositivity staining. The thin arrow indicates glomerular positivity, and the thick arrow indicates tubular positivity. (E) Histogram of LC3B immunoexpression positive area. Values are displayed as standard deviation and mean ***p < 0.001 versus control group ###*p* < 0.001 versus Colistin group. Image magnification = 100x, inset = 400x.

**Figure 8.**
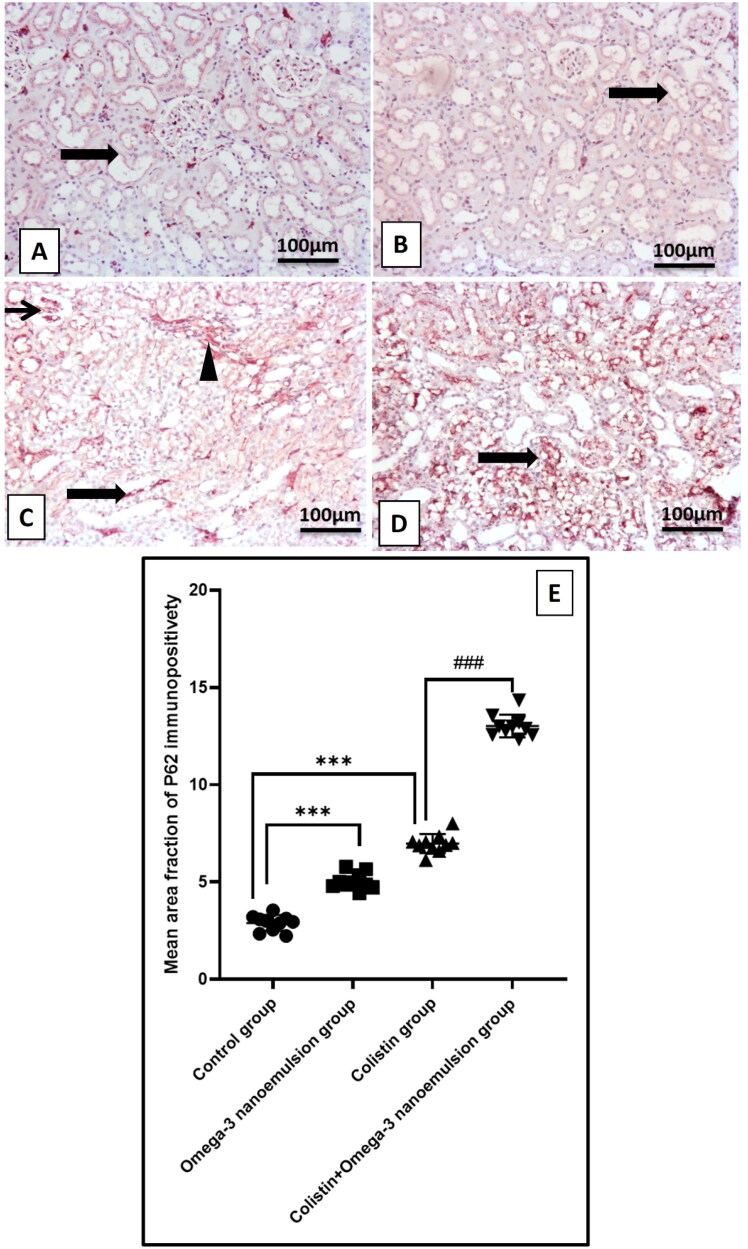
The representative IHC of p62 expression in the kidneys of different treatment groups. (A and B) The control and omega-3 nanoemulsion groups (*n* = 10) show mild to faint tubular expression. (thick arrow). (C) The colistin group (*n* = 10) shows mild to moderately strong glomerular (thin arrow), tubular (thick arrow), and interstitial immunopositive staining (arrowhead). (D) The colistin + omega-3 nanoemulsion group (*n* = 10) demonstrates high immunopositivity-stained tubular expression (thick arrows). (E) Histogram of p62 immunoexpression positive area. Values were displayed as standard deviation and mean ***p < 0.001 versus control group ###*p* < 0.001 versus colistin group. Image magnification = 100x; inset = 400x.

Figure 6 displays that colistin administration significantly p ˂ 0.001 upregulated renal mRNA of AMPK and its phosphorylated protein p-AMPK (Figure 6A,D), resulted in subsequent decrease in gene expression of mTOR, and its phosphorylated protein by Elisa assay (Figure 6B,E), concerning the control group. In contrast, concomitant administration of omega-3 nanoemulsion with colistin significantly p˂ 0.001 depressed AMPK and elevated mTOR either on the gene expression and its phosphorylated protein. Based on the inhibitory effect of omega-3 nanoemulsion on colistin-induced renal autophagy, Group IV that received both colistin and nanoemulsion showed a significant p ˂ 0.001 decrease in Elisa level of ULK1 and Beclin-1 an autophagy regulatory protein and the immunoexpression of autophagosome membrane protein LC3B (Figures 6F,G and 7D–E) with increase in the autophagy inhibition indicator p62 (Figure 8D,E) about the colistin group, which showed an increase in ULK1, Beclin-1 and LC3B with a decrease in p62 expression, respectively (Figures 6F,G, 7C,E and 8C,E). There is no significant difference between the control and negative control nanoemulsion groups (Figures 6A,B,D,E, 7A,B, and 8A,B).

### Ameliorative effect of nanoemulsion against colistin-induced renal tubular epithelial cell necroptosis

**Figure 9.**
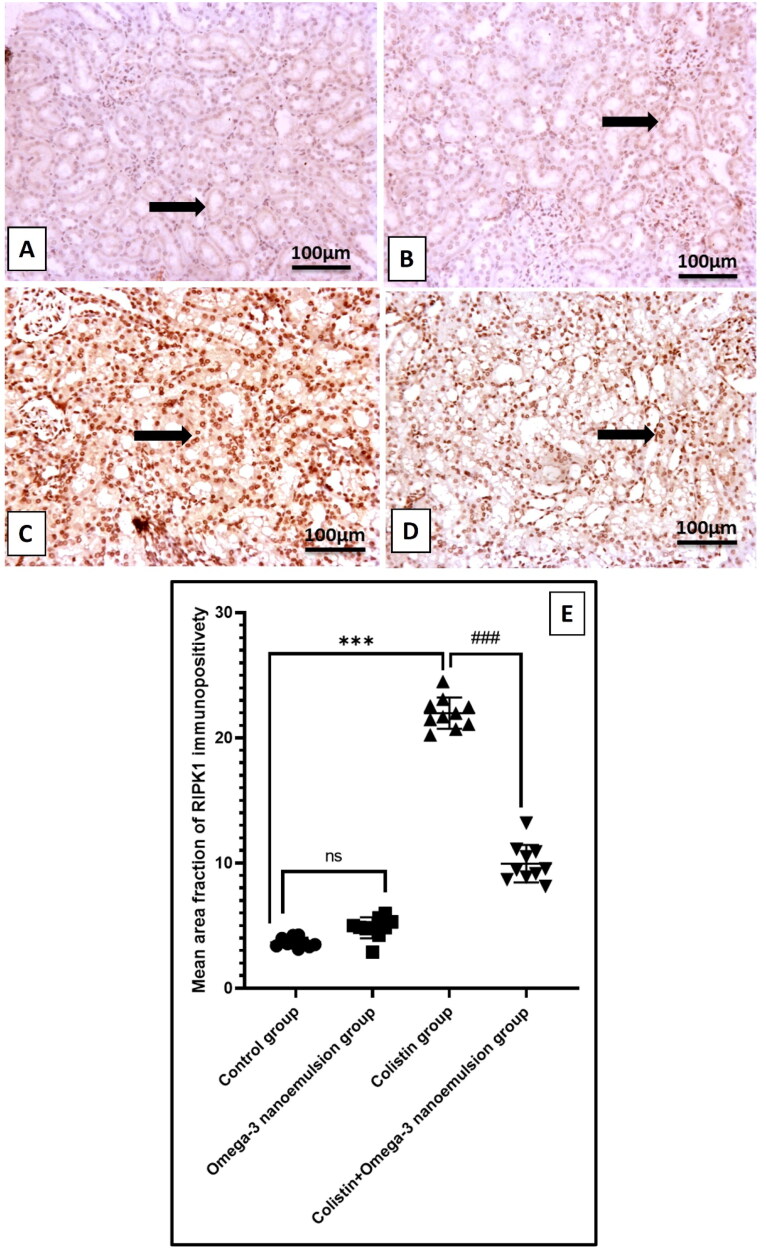
Reveals representative IHC of RIPK1 expression in the kidneys of different treatment groups. (A, B) The control and omega-3 nanoemulsion groups (*n* = 10) show faint expression of RIPK1 in tubular epithelial cells (thick arrow). (C) The colistin group (*n* = 10) reveals intense, solid expression of RIPK1 in tubular epithelial cells. (D) The colistin + omega-3 nanoemulsion group (*n* = 10) displays mild to moderate expression of RIPK1 in tubular epithelial cells. Thick arrow indicate positive immunostained cells. (E) Histogram of RIPK1 immunoexpression positive area. Values are shown as standard deviation and mean ***p < 0.001 versus control group ###*p* < 0.001 versus colistin group. Image magnification =100x, inset = 400x.

**Figure 10.**
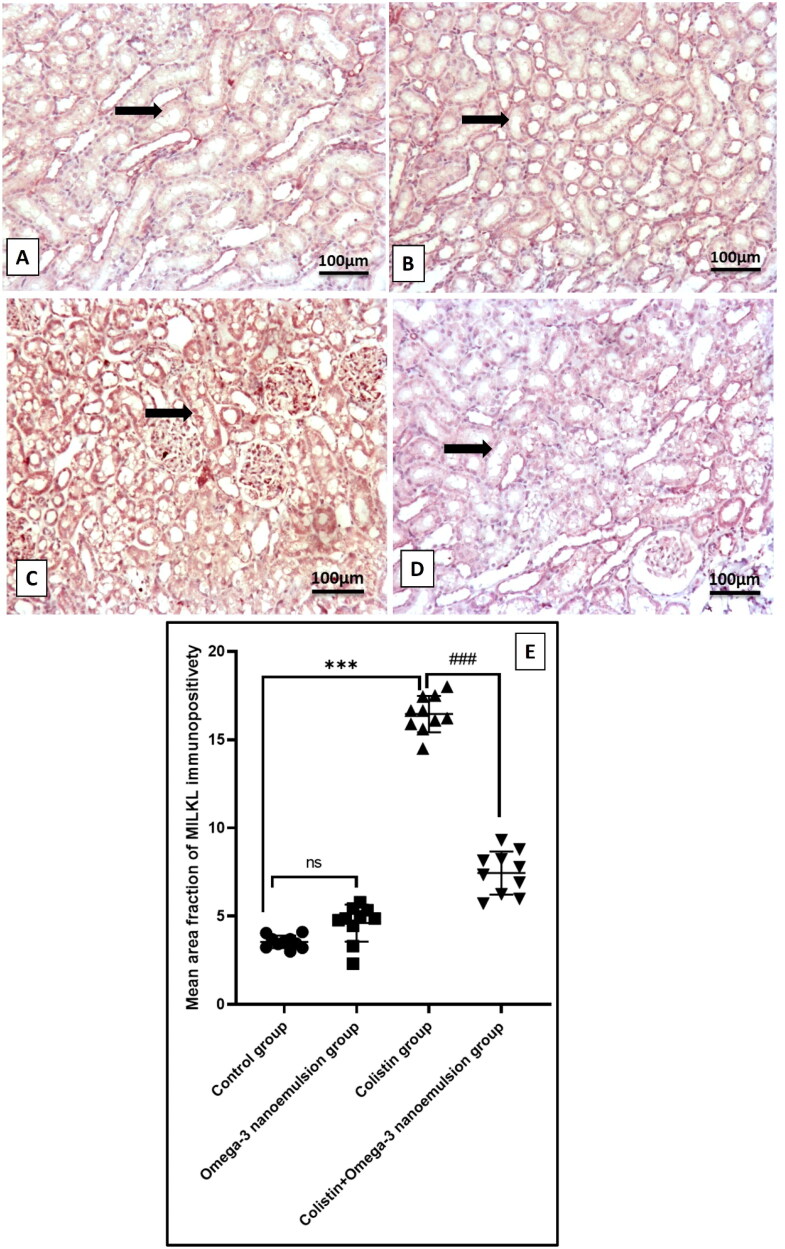
Illustrative MILKL immunohistochemistry expression in the kidneys of different treatment groups. (A, B) The control and omega-3 nanoemulsion groups (*n* = 10) display mild MILKL expression in tubular epithelial cells. (C) The colistin group (*n* = 10) reveals intense, strong expression of MILKL in tubular epithelial cells. (D) The colistin + omega-3 nanoemulsion group (*n* = 10) displays mild MILK expression in tubular epithelial cells. A thick arrow indicates positive immunostained cells. (E) Histogram of MILKL immunoexpression positive area. Values are displayed as standard deviation and mean ***p < 0.001 versus control group ###*p* < 0.001 versus colistin group. Image magnification =100x, inset = 400x.

Renal tissue necroptotic marker RIPK3 in colistin group at the mRNA level (Figure 6C) and immunoexpression of RIPK1, MILK significantly p ˂ 0.001 increased (Figures 9C,E and 10C,E) in difference to the control group. On the contrary, nanoemulsion coadministration with colistin markedly decreased necroptotic indicators RIPK3 gene expression, and RIPK1, MILKL immunoexpression (Figures 9D,E and 10D,E). There is no statistical difference between the control omega-3 nanoemulsion group (Figures 6C, 9A,B, and 10A,B).

## Discussion

Nanoemulsion of omega-3 polyunsaturated fatty acids in the lipid barrier helps its oral absorption with an increase in its half-life, protecting its chemical constituents and increasing their stability [35,36]. Particle diameter (Z-average) defines the particle size distribution. Meanwhile, the nanoemulsion’s polydispersity index (PDI) can be calculated by dividing the ratio of the standard deviation by the mean droplet size, measuring the polydispersity. Meanwhile, it indicates the quality and homogeneity of the nanoemulsion. In Figure 1C, the result revealed that the mean of the hemodynamics of the dispersion diameter is 150.4. This means that the nanoemulsion particle size is desirable, as the accepted size of nanoparticles is ≤500 nm [37]. The results of the current study are supported by another study by Galdino de Souza et al. [38], who documented a similar-sized vegetable oil-based nanoemulsion using an ultrasonic technique. The PDI value ranges from 0 to 1, where 0 means monodispersable, while 1 indicates the dispersion of polydispersable particles [39]. PDI with a mean less than 0.2 is desirable as it suggests that the nanoemulsion particles are similar in size and not aggregates [40]. In the present study, the PDI mean value is 0.21 nm, which means that the PDI of the nanoemulsion particle is within the desirable range. Zeta potential usually indicates dispersion stability; its value can be affected by several factors, including the presence of electrolytes, nanoemulsion phytochemical characterization, and their adsorption ability [41]. Zeta potential power indicates suspension particle stability; in this context, molecules resist any suspension with relatively high positive or negative zeta potential, avoiding aggregation and increasing suspension stability. When the zeta potential value is zero, a surface charge is not found, which increases particle interaction and helps its aggregation. Generally, suspension with zeta potential values of more than −30 mV is considered stable [42]. The current study (Figure 1D) revealed that the mean value of Zeta potential dispersion is −24 mV, which indicates its homogeneity. This finding parallels the survey conducted by Galdino de Souza et al. [38], who reported that the zeta potential dispersion means the value of the fish oil nanoemulsion is −16.9 mV.

The present research included two main findings. The first one revealed that omega-3 nanoemulsion improved colistin-induced renal impairment with improvements in renal histology. The second one focused on the ameliorative mechanistic role of omega-3 nanoemulsion against colistin-induced nephrotoxicity, and it halted renal oxidative insults, renal inflammation, counteracted colistin-induced renal autophagy, and mediated necroptosis, preserving renal tubular epithelial cell health. These results provide novelty in treating colistin-induced nephrotoxicity with an omega-3 nanoemulsion and a declaration of its mechanistic role. It is well established that oxidative stress is a critical factor in the pathophysiology of nephrotoxicity caused by colistin, which exhibits an imbalance between prooxidant and antioxidant system production [43]. Reactive oxygen species (ROS) (e.g., hydrogen peroxide (H_2_O_2_), superoxide anion radical O_2_^-^, nitric oxide (NO), and hydroxyl radical (OH)) accumulation into the cytosol leads to further damage in cellular macromolecules, including endoplasmic reticulum stress, mitochondrial dysfunction, membrane lipid bilayer disintegration, lipid peroxidation, and damage to DNA [44]. Consistently, the present investigation results revealed colistin-induced remarkable oxidative insult in the significant elevation of lipid peroxidation marker and decreased the antioxidant enzymes catalase and superoxide dismutase with a reduced total antioxidant capacity (TAC) level compared to the control group. This finding is consistent with the previous studies conducted by [45,46]

On the other hand, omega-3 nanoemulsion oral intake with colistin injection exhibited antioxidant properties by reducing MDA and elevating SOD, CAT, and TAC, following the previous work performed by Hashim et al. [47]. Inflammation plays a leading role in colistin-induced renal injury [48]. In this investigation, intraperitoneal injection of colistin induced a severe inflammatory cascade that increased renal supernatant levels of TNF-α, IL-6, and IL-1β proinflammatory markers by ELISA assessment concerning the control group. In contrast, omega-3 nanoemulsion ingestion significantly (p ˂ 0.001) reversed inflammatory marker levels to near normal, combating renal inflammation. These results parallel the previous work conducted by Hussein et al. [31] on the documented anti-inflammatory effect of eicosatetraenoic acid-loaded silica nanoemulsion against DEN-induced hepatic inflammation by reducing the activity of TNF-ά, IL6, and IL1. Therefore, the protective effect of nanoemulsion on tubular degeneration and preservation of tubular epithelium may be attributed to its antioxidant and anti-inflammatory character.

It is well known that the basal autophagy process is required for cell survival and physiology. It plays a beneficial role in preserving the health of proximal tubular epithelial cells, podocytes, glomerular endothelial cells, and glomerular mesangial cells. It helps remove cellular debris, damaged organelles, and cellular energy production [49]. The most common type of autophagy is macroautophagy, in which cells surround damaged organelles with a double layer of membrane called autophagosomes, which fuse with lysosomes to form autolysosomes, recycling them to produce energy [50,51]. Multiple kinases are involved in the regulation of the autophagic pathway, among them, adenosine monophosphate (AMP)-activated protein kinase (AMPK), which is a solid stimulator for the autophagy process either by inactivation of rapamycin (mTOR) [52], moreover, through its role in the activation of phosphorylation of ULK1 [53]. The activated ULK1 phosphorylates Beclin-1, considered as one of the leading indicators of cellular autophagy [54]. Beclin-1 binds to another cofactor to form a BCL2-interacting coiled-coil protein (BECN) complex, which promotes autophagosome formation [55]. LC3 binds to autophagosomes, followed by the LC3-1-to-LC3-II conversation. Therefore, LC3-II was considered the primary indicator for autophagic flux [56]. It was reported that there is a strong relationship between the accumulation of ROS and autophagy induction, as ROS overproduction irreversibly damages DNA and oxidizes cellular biomolecules, which activates the autophagy process to clean the cell from damaged DNA, proteins, and lipids [57].

The results of the present study reported that colistin injection induces renal autophagy by a significant enhancement in the immunoexpression of the LC3B and protein level of ULK1, and Beclin-1, with a marked decrease in the expression of p62, as a result of increased gene expression and phosphorylated protein of the autophagic regulator kinase AMPK and a decline in the gene expression and phosphorylated protein of mTOR. It can be attributed to colistin-induced oxidative stress, which is considered one of the positive regulators of the autophagic process. The current investigation’s finding parallels the previous study performed by Dai et al. [4], who reported that 7.5 mg/kg of colistin increases the level of renal autophagic biomarkers LC3B and Beclin-1. Moreover, the study conducted by Aksu et al. [58] documented that colistin administration increased testicular autophagy *via* its increase in LC3B expression level. Interestingly, treatment of colistin rats with omega-3 nanoemulsion reversed the autophagic process by a decrease in LC3B, ULK1, and Beclin-1 with an increase in p62 immunoexpressions, resulting in modulation of autophagic regulator genes by a reduction of AMPK and an increase in mTOR gene expressions and protein level concerning the colistin group. This finding aligns with the previous study performed by [59]. Omega-3 nanoemulsion exhibited an anti-autophagy character that downregulated pathological autophagy; this can be regarded as its antioxidant power antagonizing the autophagic process.

Necroptosis can be defined as a preprogrammed cell death like apoptosis and is morphologically characterized by organelle dysfunction, cellular swelling, and plasma membrane rupture-like necrosis [60,61]. Necroptosis was considered the regulated form of necrosis that was recently investigated [62]. Several kinases control the necroptosis process, such as RIPK-1, RIPK-3, and MLKL [63,64]. Triggering of necroptosis can be performed by several exogenous and endogenous stimuli, such as toll-like receptors, especially TLR3/4 [65]. In addition, several necroptosis stimulator factors, including TNF-α, were considered the most triggering factors of necroptosis [66]. It is well known that necroptosis plays a pivotal role in the pathogenesis of acute kidney injury [64]. Following the present work, colistin intraperitoneal injection induces a marked increase in the level of necrosomes by a significant increase in the immunoexpression of necroptosis-related proteins RIPK-1 and MLKL, with elevated mRNA expression of RIPK-3. The upregulatory effect of colistin on renal tubular epithelial cell necroptosis can be regarded as its stimulatory effect on the production of inflammatory markers, especially TNF-α, which is considered the main initiator of the necroptosis process. Fortunately, omega-3 nanoemulsion displayed a protective effect against colistin-induced necroptosis by decreasing immunoexpression and gene expression of necroptosis-related proteins RIPK-1, MLKL, and RIPK-3. The protective effect of nanoemulsion in combating colistin-induced necroptosis is regarded as its anti-inflammatory effect, with a decrease in the renal supernatant of TNF-α, a principal regulator of the necroptosis process. This is the first study that discusses the necroptotic effect of colistin as a new pathologic theory and the protective role of omega-2 polyunsaturated fatty acid nanoemulsion on it.

**Figure 11.**
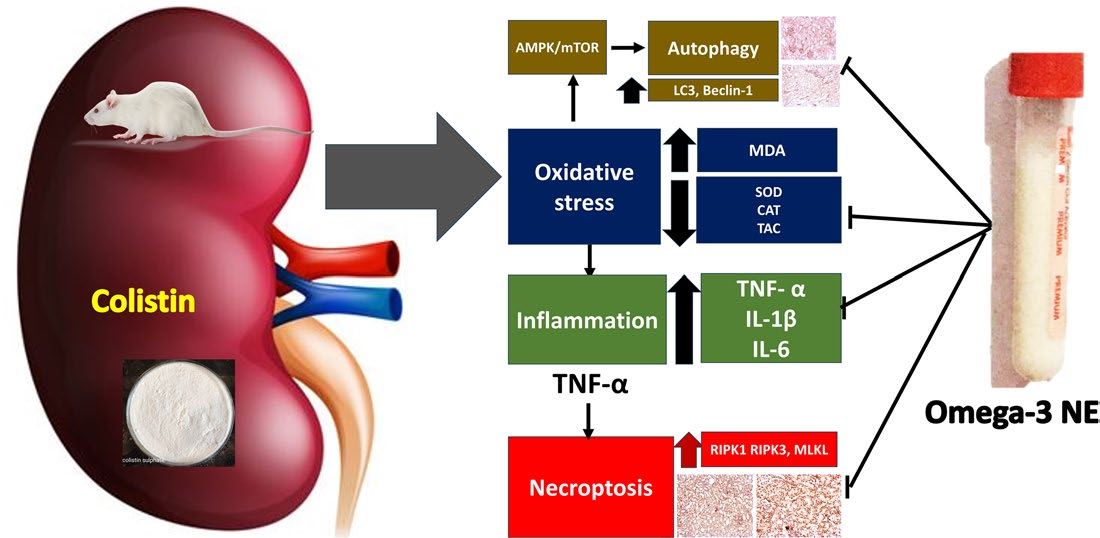
The mechanisms by which colistin induces nephrotoxicity involve complex interactions, and the inhibitory role of omega-3 nanoemulsion is significant. The primary mechanism of nephrotoxicity is oxidative stress, which stimulates the inflammatory Cascade, resulting in TNF-α potentiating the necroptotic process. Furthermore, oxidative stress stimulates pathological autophagy. The combined effects of stimulated inflammation, necroptosis, and excessive autophagy lead to renal tubular epithelial cell death, histopathological alterations, and deterioration of renal function.

Colistin intraperitoneal administration significantly deteriorates renal functions, creatinine, and BUN and increases 24-h urinary protein and KIM-1 with marked decrease in creatinine clearance. The jeopardy of kidney function and morphological changes such as tubular necrosis, degeneration are associated with severe interstitial inflammation. These findings are equivalent to what was reported by Ghlissi et al. [67], who documented seven days of treatment with colistin elevation in the plasma level of creatinine and KIM-1, which is considered a kidney injury indicator, associated with tubular epithelial necrosis, tubular dilation, and vacuolation with numerous markers. On the contrary, omega-3 nanoemulsion coadministration with colistin produces remarkable renal function improvement with subsequent renal histology improvement. The nephroprotective effect of omega-3 nanoemulsion against colistin-induced nephrotoxicity is regarded for its antioxidant, anti-inflammatory, anti-necroptosis, and anti-pathological autophagy (Figure 11). This study face limitation to investigate the pharmacokinetics of the omega-3 nanoemulsion, we will consider this valuable point in our future studies.

## Conclusions

This study discussed the ameliorative outcome of omega-3 nanoemulsion against colistin-induced renal toxicity. Omega-3 nanoemulsion markedly improves renal function with restoration of renal histology *via* its antioxidant and anti-inflammatory effects and its modulation of colistin-induced autophagy and necroptosis. Omega-3 nanoemulsion halts pathological autophagy occurrence and has anti-inflammatory effects, especially its reduction to TNF-α, stopping its initiation in the renal necroptotic process (Figure 11).

## Data Availability

All authors declare that the data supporting the findings of this study are available within the paper.
